# Oral health of patients with epidermolysis bullosa compared to healthy controls – a retrospective study from a specialized center

**DOI:** 10.1186/s12903-025-07457-2

**Published:** 2025-12-05

**Authors:** Tara Groß, Sophia Aurelia Stocker, Konstantin J. Scholz, Cristina Has, Kirstin Vach, Fabian Cieplik

**Affiliations:** 1 Department of Operative Dentistry and Periodontology, Center for Dental Medicine, Faculty of Medicine, Medical Center, University of Freiburg, Hugstetter Str. 55, 79106 Freiburg, Germany; 2 Institute of Medical Biometry and Statistics, Faculty of Medicine, Medical Center - University of Freiburg, Freiburg, Germany; 3 Department of Conservative Dentistry and Periodontology, University Hospital Regensburg, Franz-Josef-Strauß-Allee 11, Regensburg, 93053 Germany; 4 Department of Dermatology, Medical Center, University of Freiburg, Freiburg, Germany

**Keywords:** Epidermolysis bullosa, Oral health, Dental treatment, Tooth extractions, Retrospective study, General anesthesia

## Abstract

**Background:**

Epidermolysis bullosa (EB) is a rare genetic disorder characterized by skin and mucosal fragility, leading to significant oral health challenges due to mucosal blisters, pain, up to microstomia and ankyloglossia in severe cases. This retrospective study aimed to assess oral health in EB patients compared to a healthy control group to develop tailored dental protocols.

**Methods:**

Medical records and panoramic x-rays of EB patients (*n* = 40) treated at the Center for Dental Medicine, University Medical Center Freiburg (2014–2024), were analyzed and compared to a healthy control group (*n* = 40) with similar age- and gender distribution. Oral health was evaluated using DMFT/dmft index and treatment data including extractions and fillings. Statistical analysis was performed using the Wilcoxon-Mann-Whitney test (α = 0.05).

**Results:**

37 EB patients (median (25–75%) age: 10 (5;17) years) and 37 controls (median age: 9 (5;16) years) were included after three exclusions each. EB patients exhibited significantly higher DMFT/dmft scores (*p* < 0.0001), more carious teeth (*p* < 0.0001), resulting in more tooth extractions (*p* < 0.0001) compared to the control group.

**Conclusions:**

EB patients had significantly higher caries burden and required more extractions compared to controls. The findings of this study highlight the necessity of specific dental interventions emphasizing preventive care.

## Introduction

With an overall prevalence of 1:18,500 in Germany across all subtypes [1], epidermolysis bullosa (EB) is a rare genetic skin disorder which is classified into four main types: epidermolysis bullosa simplex (EBS), epidermolysis bullosa junctionalis (JEB), epidermolysis bullosa dystrophica (DEB) and Kindler epidermolysis bullosa (KEB) and more than 30 subtypes [2]. The main types and various additional subtypes differ in terms of their etiology, inheritance, and clinical course. Common to all EB types is the genetically determined detachment within different layers of the dermal-epidermal junction, which leads to blistering [3].

EBS, the mildest and most common form of EB with a reported worldwide prevalence of 1:85,000 to 1:500,000 [4], is usually inherited in an autosomal dominant manner and is caused by a separation within the basal layer of keratinocytes [5]. The autosomal recessive JEB is caused by a separation of the lamina lucida of the basement membrane zone of the skin. One of the phenotypic oral manifestations of JEB is hypoplasia of dental enamel [6]. DEB, the most severe form of EB, is inherited in a dominant (DDEB) or recessive (RDEB) manner and is associated with mutations in the gene coding for the major anchoring protein collagen VII, i.e., *COL7A1* [7]. Therefore, DEB is characterized by a splitting of the skin in the sublamina densa [8]. Blistering in DEB cases leads to partly severe scarring of the skin and mucosa [7, 9, 10]. DEB also manifests itself in the oral cavity of those affected in varying degrees of severity and diversity [11]. The accompanying symptoms of the disease, such as the limitation in mouth opening (microstomia), ankyloglossia, i.e. the adhesion of the tongue to the base of the mouth [12], fusion of the fingers because of the gradual progression of scarring, and pain and blistering when brushing teeth severely restrict the patient’s ability to maintain sufficient oral hygiene [9, 11]. Unfortunately, for the same reasons direct restorative treatments aiming for tooth preservation such as dental fillings, root canal treatments or even intraoral x-ray diagnostics are difficult and often impossible to implement, especially for posterior teeth due to the difficult accessibility. As a result, EB is often characterized by severely damaged, rapidly progressing tooth decay and serial tooth extractions at a young age [13, 14]. This enormously reduces the quality of life in addition to the restrictions of underlying disease [15, 16]. However, specific information about the oral health related quality of life is scarce for EB patients [17].

In the last 20 years, there have generally been very few studies on the oral health of EB patients, especially hardly any comparisons with healthy patient groups [17, 18]. In terms of tooth extraction frequency, there are mainly case reports with small numbers of patients [19–21].

However, there appears to be a lack of holistic dental treatment concepts. A recent guideline on oral manifestations and clinical management has proposed recommendations for dental therapy and listed specific EB centers (Krämer et al., 2020) [11]. Given that the Medical Center of the University of Freiburg serves as a specialized EB center, the objective of the present study was to retrospectively evaluate the oral health status of a cohort of EB patients treated at this institution and to compare the dental treatments they received with those provided to a control group of healthy individuals. The null-hypothesis of the study was that the oral health of patients with dental treatment needs with Epidermolysis bullosa does not differ significantly from a generally healthy control group with similar age- and gender distribution.

## Materials and methods

### Study design

The present study was designed as a retrospective case-control study. The objective was to assess oral health in EB patients compared to a healthy control group treated at the Center for Dental Medicine at the University Medical Center Freiburg, to develop tailored dental protocols. The data of all patients was handled with the utmost care and was pseudonymized so that no traceability is possible. Additionally, informed and written consent was obtained from all patients from whom images or x-rays were published. The study design was approved by the institutional review board of the Albert-Ludwigs-University of Freiburg, Germany (Number: 24–1317-S1-retro) in accordance with the 1964 Helsinki Declaration and its later amendments and comparable ethical standards. The study was registered at the German Clinical Trials Register (DRKS 00033832, 2024-10-11, FRKS 005182) and meets the requirements of the STROBE checklist (STrengthening the Reporting of OBservational studies in Epidemiology) for case-control studies [22].

### Patient cohort

In total, medical records of 40 EB patients who received dental treatment in general anesthesia with intubation at the Center for Dental Medicine of the Medical Center of the University of Freiburg between 01.01.2014 and 31.12.2024 were examined in this study. In the EB group, all available types were included (EBS *n* = 3, JEB *n* = 5, DEB *n* = 31 and EB unknown *n* = 1). Medical records and pre- and postoperative x-rays (orthopantomograms) were analyzed and compared to a healthy control group. All data were collected independently by two experienced dentists (more than three years of professional experience). In case of dissonance, the respective instances were resolved through discussion. Based on these 40 patients in the test group, 40 patients with similar age and gender distribution were systematically selected from the patient’s charts and included in this study. Due to insufficient documentation (specifically, incomplete or entirely missing records regarding the number of fillings and tooth extractions) for three patients with EB, these individuals, along with three age- and gender-matched patients from the control group, were excluded from the data analysis.

### Dental status and performed treatments

Along with collected population data, such as gender, age and type of EB, the oral health status was determined using the DMFT/dmft index (DMFT - mixed or permanent dentitions, dmft - primary dentition) [23]. The number of decayed (d; D), missing (m; M) and filled (f; F) teeth were calculated. The DMFT/dmft can reach a maximum score of 28,because it accounts for the 28 permanent teeth, excluding the third molars (wisdom teeth) [24]. Furthermore, characteristic accompanying symptoms of EB like ankyloglossia and microstomia were determined from the patient anamnesis charts (dichotomous classification). In addition, the oral hygiene within the EB and the control group was categorized as good, medium or poor and taken from the available medical documentation. According to the typical procedure of the department where the treatments were carried out, good oral hygiene corresponds to an approximal plaque index (API) [25] of 25–40% and sulcus bleeding index (SBI) [26] < 10%, medium oral hygiene corresponds to an API >40–70% and SBI ≥ 10%−50%, and poor oral hygiene corresponds to API >70% and SBI >50–100%, whereby the worse of the two values was used for classification in each case.

In 7 cases patients had to undergo more than one treatment in intubation anesthesia during the observation period, in one case up to 4 times. For EB patients who required multiple dental treatments under general anesthesia within the observation period, the dental status from the first appointment was used.

### Data analysis

For a descriptive analysis, medians with 25%/75% percentiles, and relative and absolute frequencies were calculated. The level of significance was set to 0.05. Scatter plots were used for graphical presentation. Due to the skewed distribution Wilcoxon-Mann-Whitney tests were used to test for group differences of continuous data. Fishers exact test was used for group comparisons of categorical data. Due to the pilot character of the present study no correction for multiple testing was performed. All computations were done with STATA (Version 17.0, CollegeStation, TX, USA).

## Results

### Patient cohort

The age of the patients ranged from 3 to 43 years, with a median age of 9.5 (5;17) years (EB: 10 (5;17) years, C: 9 (5;16) years). The EB group as well as the healthy control group consisted of 20 female and 17 male patients each (Table 1).

**Fig. 1:**
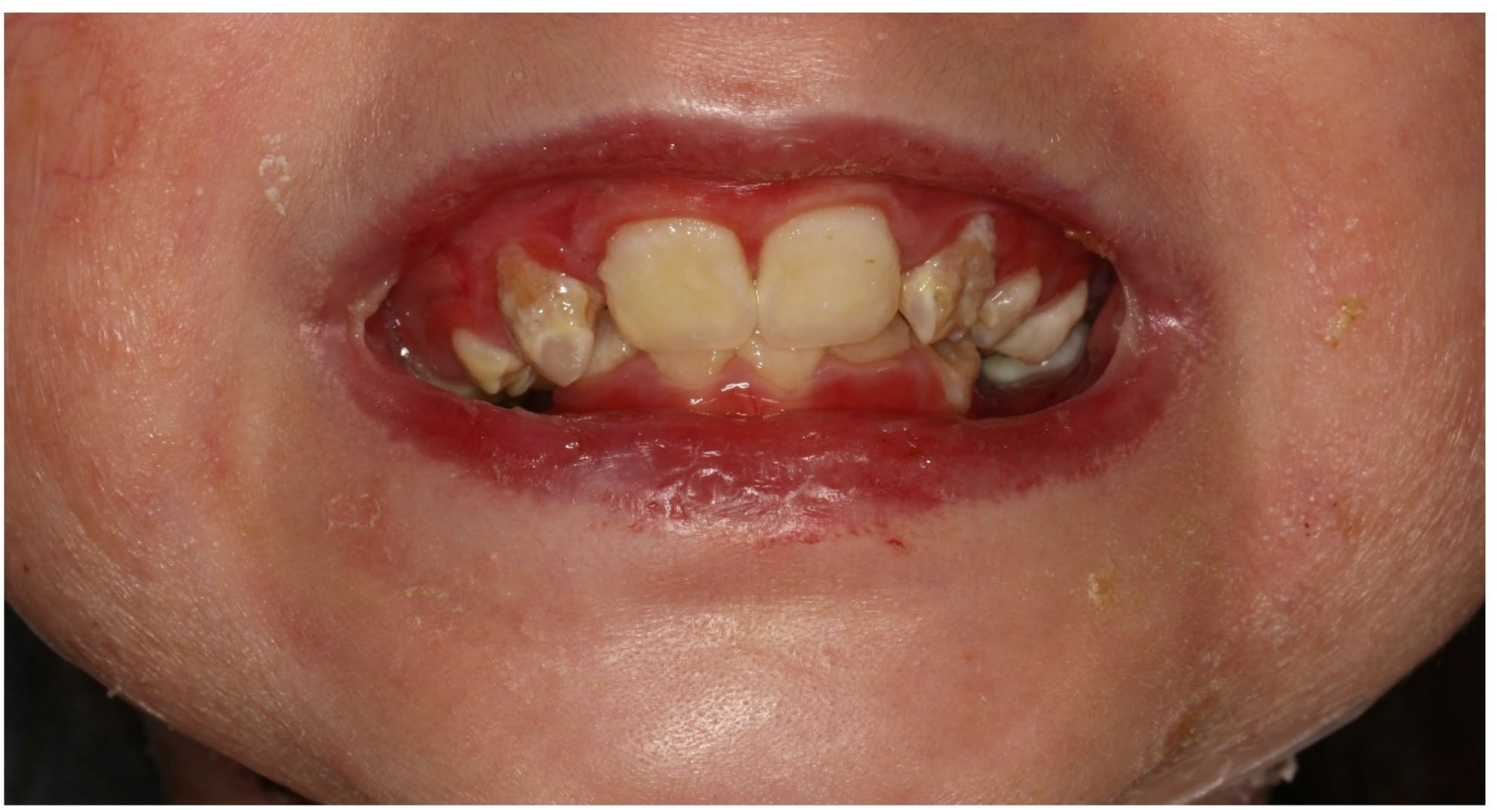
Exemplary picture of a DEB patient (7 years old, female), fragile extra-oral skin, intra- and peri-oral blistering. Alterations in dental enamel, indicative of hypoplasia, are visible. There is a noticeable plaque accumulation, likely accompanied by marginal gingival inflammation. The lip skin appears reddened and bears marks of previous scarring. Even with minimal mouth opening, the lips exhibit circular tension, suggesting microstomia

Within the EB group, DEB was the most frequent subtype in the examined patients (75.7%). In 64.3% of DEB cases, a recessive form (RDEB) was documented. The other DEB cases were not differentiated further, so no case of DDEB was documented. Patients with JEB accounted for 13.5% and EB simplex for 8.1%, leaving one patient having an unspecified EB diagnosis (2.7%). Of the EB patients listed, a total of 6 had to be treated more than once under general anesthesia, including one patient who required 4 dental treatments in general anesthesia with intubation during the observation time. Figure 1 shows an exemplary picture of a DEB patient.

**Table 1:** Overview of the patient cohort, rows showing EB-group (left) and healthy control group (right), columns presenting age specified in median (25%; 75% percentiles) and sex in percentages

	EB group (*n* = 37)	Healthy control group (*n* = 37)
Age	10 (5;17) years	9 (5;16) years
Sex	20 female (54%) 17 male (46%)	20 female (54%) 17 male (46%)

### Dental status

The parameters examined in this study are summarized in Table 2. There were no significant differences among groups regarding tooth count and filled teeth (initial fillings) at first assessment. The EB group showed significantly more carious teeth and higher DMFT/dmft (both *p* < 0.0001) as compared to the healthy control group. Furthermore, EB patients received significantly more extractions during therapy than patients in the healthy control group (*p* < 0.0001).

**Table 2:** Overview of the distribution of the dental status and performed treatments for EB patients and the healthy control group. Rows categorized in EB group, healthy control group and p-value. Columns showing tooth count, decayed teeth, initial fillings (already filled teeth at first assessment), DMFT/dmft, extracted teeth during therapy and oral hygiene status; all specified in median (25%; 75% percentiles) and oral hygiene displayed in percentages

	EB group (*n* = 37)	Healthy control group (*n* = 37)	*p*-value
Tooth count p50 (p25; p75)	23 (20; 26)	24 (20; 28)	*p* = 0.0842
Decayed teeth p50 (p25; p75)	11 (7; 15)	0 (0; 0)	*p* < 0.0001
Initial fillings p50 (p25; p75)	2 (0; 6)	2 (0; 5)	*p* = 0.9799
DMFT/dmft p50 (p25; p75)	15 (11; 20)	3 (0; 6)	*p* < 0.0001
Extracted teeth during therapy p50 (p25; p75)	4 (2; 9)	0 (0; 0)	*p* < 0.0001
Oral hygiene status	Good: 28% Medium: 20% Poor: 52%	Good: 36.4% Medium: 40.9% Poor: 22.7%	*p* = 0.107

Descriptively, it was noted that 32% of the existing teeth of EB patients required extraction during therapy (an average of 6.9 teeth), compared to only 1% of the teeth in the healthy control group. In one EB case, all teeth had to be completely removed. The highest number of extracted teeth was 23, leading to only three residual teeth in the 17-year-old DEB patient.

When subdividing the EB group into patients ≤ 10 years (*n* = 19) and > 10 years (*n* = 18), DMFT/dmft of 15 (11;18) and 16.5 (11;21) by median were observed, respectively. Among those under 10 years old, a total of 57% of teeth exhibited carious lesions with treatment need, whereas among those over 10 years old, 45% were carious.

**Fig. 2:**
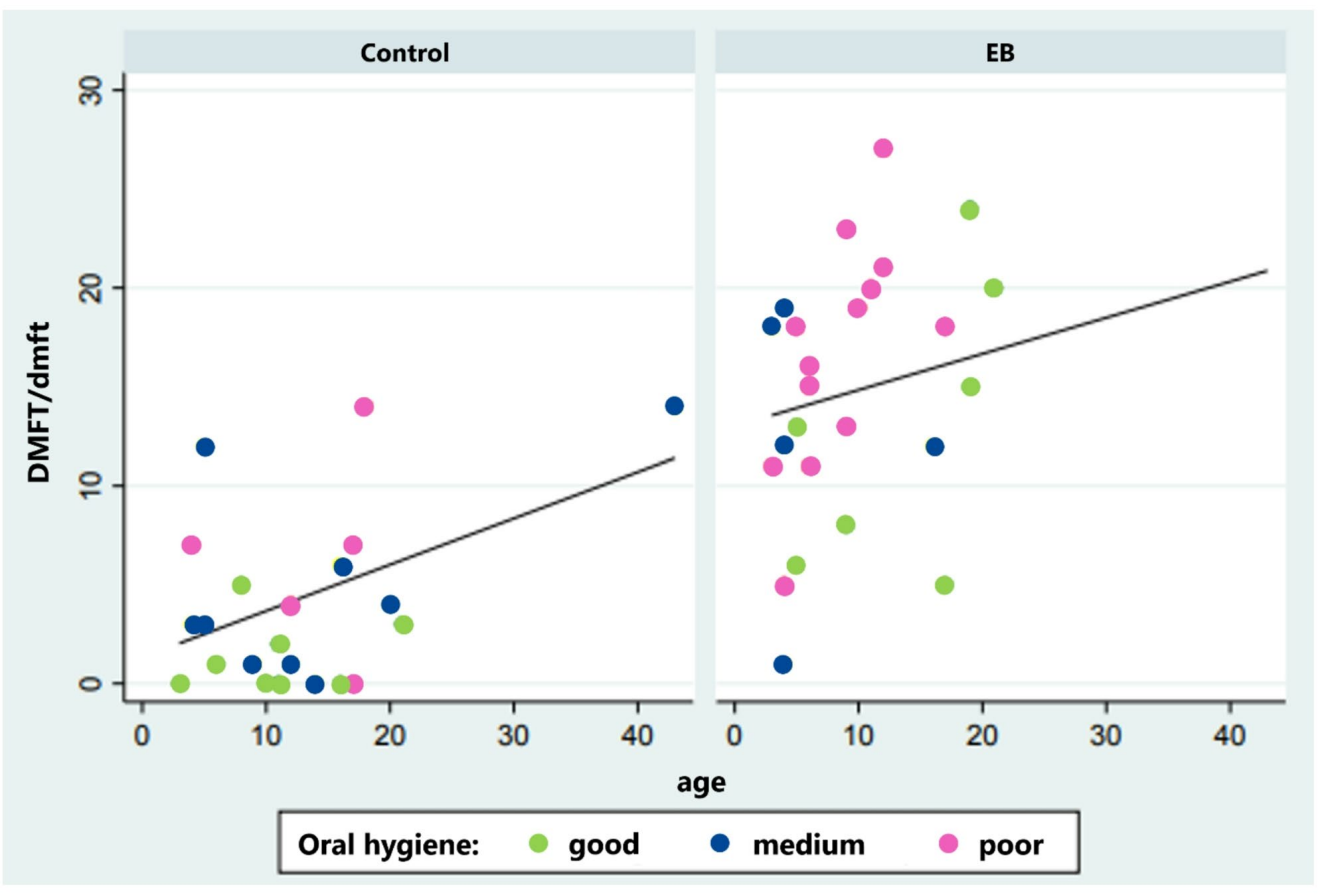
Association between age and DMFT/dmft index for the test group (EB) and the control group according to oral hygiene status. The x-axis represents the patients’ age (in years), and the y-axis shows the DMFT/dmft score, indicating the total number of decayed (D/d), missing (M/m), and filled (F/f) teeth. Colors indicate the recorded oral hygiene categories: green = good, blue = medium, and pink = poor. For visualization the linear regression line showing the association of age and DMFT/dmft within each group was presented

Furthermore, 52% of EB patients and 22.7% of the control group exhibited poor oral hygiene. Additionally, patients from both groups with poor oral hygiene had more fillings placed (initial fillings) (median: 5 (0;8)), and more fillings were performed during the single treatment under anesthesia (median: 8 (5;15)) in comparison to patients with a good oral hygiene, who had a median of 1 (0;3) filling initially and 5 (3;8) fillings placed during treatment. The scatter plots visualizing the relation between the DMFT/dmft and age, show for both groups a tendency to a higher DMFT/dmft with increasing age with larger DMFT/dmft values for EB patients with poor oral hygiene (Fig. 2). When further comparing the distribution of oral hygiene status between the EB and control group a higher prevalence of poor oral hygiene in EB patients was observed, even though this did not reach the level of statistical significance (*p* = 0.107).

Regarding microstomia in the EB group, three patients had no restrictions according to the available documentation, no information was available for 11 patients, and 25 patients with DEB and one patient with EBS exhibited microstomia. There were reports of ankyloglossia in 8 of the examined patients, all of whom belonged to the DEB type.

**Fig. 3:**
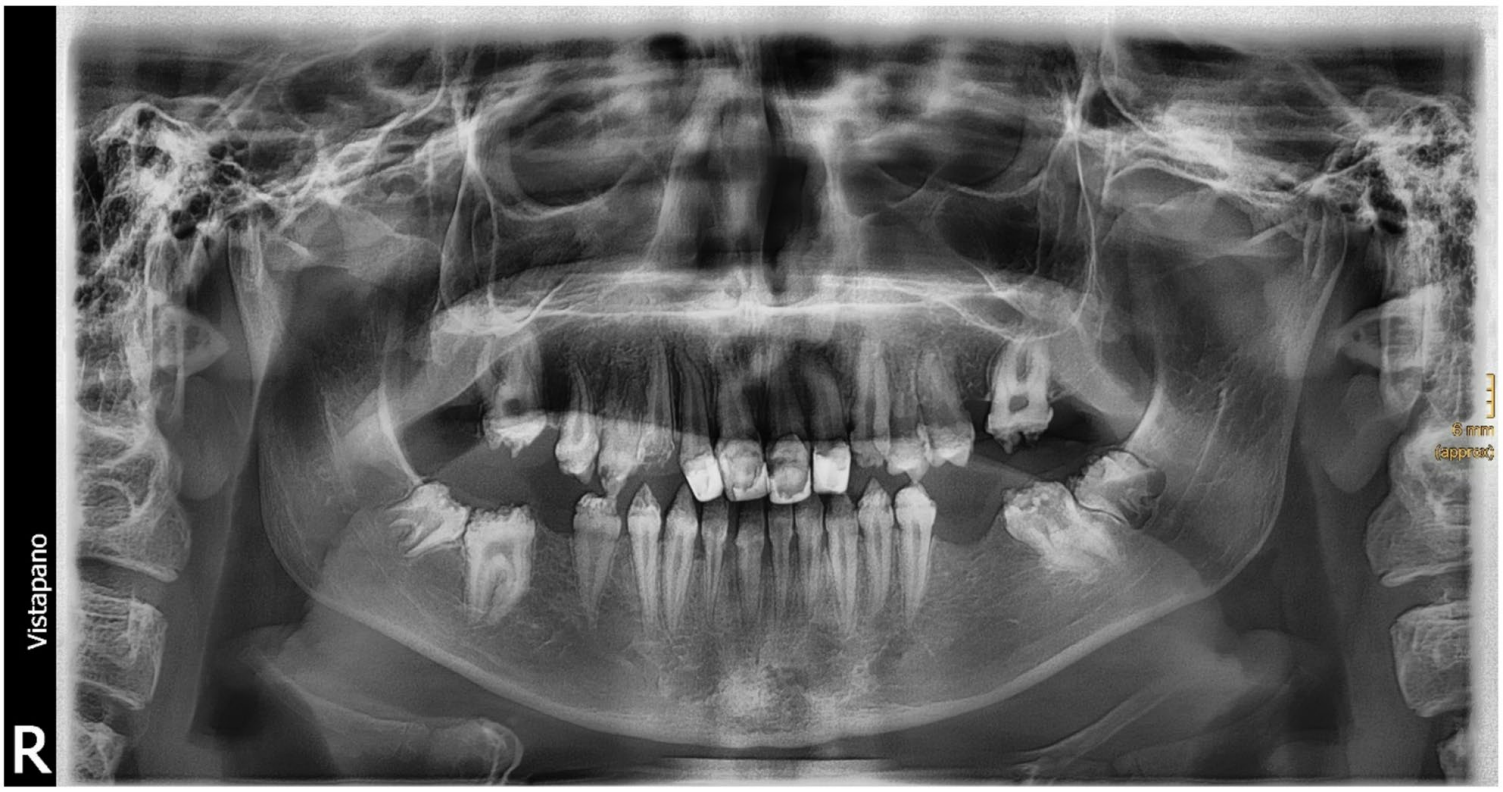
Panoramic x-ray of a 14-year-old male JEB patient, presenting generalized severe enamel hypoplasia. Enamel alterations are also visible in non-erupted teeth 37, 38, 47, 48. Upper incisors revealed extensive restorations

**Fig. 4:**
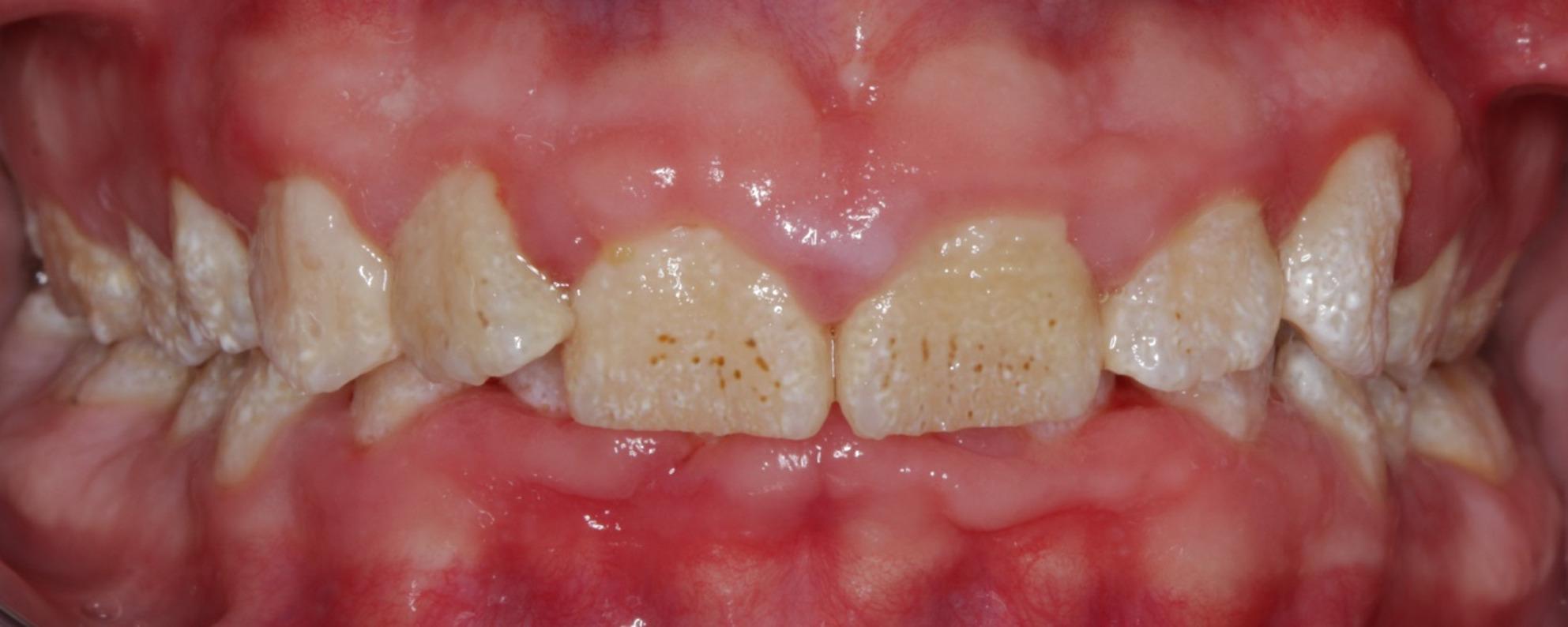
Exemplary picture of a JEB patient (14-year-old, male) with generalized enamel hypoplasia

According to documentation and x-ray analysis, all three patients with JEB included in this study exhibited radiographically and clinically visible enamel hypoplasia. However, the clinically diagnosed hypoplasia was not genetically tested for a possible diagnosis such as amelogenesis imperfecta. Figure 3 shows an example of a panoramic X-ray of a JEB patient (14.7 years). Figure 4 shows a typical clinical picture of a JEB patient (14.9 years).

## Discussion

The findings of this study significantly highlight disparities between the patients diagnosed with EB and the control group, underscoring the unique challenges faced by EB patients in maintaining oral health [11]. Hence, the stated null-hypothesis that the oral health of EB patients does not differ significantly from healthy patients could be rejected by our study. The majority of EB type treated during the observation period was DEB which is in alignment with other reports that patients with DEB, especially patients suffering from RDEB showed the most severe oral manifestations and consequently had a higher need for dental treatment [27].

Looking at the initial situations in the EB group and the control group, no significant differences were found regarding tooth count and fillings before dental treatment. However, DMFT/dmft, numbers of carious teeth and performed tooth extractions during therapy revealed statistically significant differences between the EB group and the healthy control group. This indicates a markedly higher caries experience among EB patients (Krämer et al., 2023) [28]. The high DMFT/dmft score and numbers of carious teeth in the EB group can be attributed to the chronic nature of EB, which affects the oral mucosa, leading to difficulties in maintaining proper daily oral hygiene [8]. Additionally, restricted mouth opening (microstomia), and the presence of ankyloglossia of DEB patients contributed to inadequate oral care [11], thus increasing the risk of dental caries.

When examining the DMFT/dmft in relation to age, both groups exhibited a linear relationship. However, the DMFT/dmft values for EB patients were consistently higher across all age groups. This suggests that EB patients accumulate tooth decay, e.g. due to dental caries at a higher rate [8] and from an early age, likely due to the aforementioned challenges in maintaining oral hygiene and a lack of the education of parents and patients about nutrition and caries prevention, regular check-ups [19, 29, 30] and fluoridation [19]. However, more effective acidic fluoride supplements can be painful when blisters and ulcers are present, and patients may therefore choose not to use them [14, 31]. The subgroup analysis of patients ≤ 10 years and >10 years showed DMFT/dmft scores with a median (25%−75%) of 15 (11;18) and 16.5 (11;21), respectively. This further underscores the progressive nature of dental issues in EB patients as they age. An observational and retrospective uncontrolled study based on the analysis of medical records of 278 EB patients in a tertiary hospital revealed dental alterations in 36% (100 patients) and possibly related malnutrition in 13% of the patients [32]. Out of these 100 patients with dental alterations, the authors observed that 54 (54%) were RDEB, 28 (28%) were DDEB, 13 (13%) were EBS, three (3%) were JEB, two (2%) were KEB, whereas none was pruriginous DEB. Irregularities in enamel in terms of defects, irregularities or hypoplasia as visible in the x-ray of Fig. 3 as well as atypical early restorations were also described by other authors [32].

In line with the extent of carious lesions in EB patients, our study shows that EB patients exhibited a by tendency poorer oral hygiene than the control group, even if not significant. Poor oral hygiene in EB patients may strongly be associated with the other symptoms of the disease, i.e. pain during oral care routines up to restricted mouth opening and ankyloglossia. Nevertheless, Krämer et al. stated that every patient with EB can perform oral hygiene, implied that all the information and possible aids are provided [11]. Another possible explanation of a higher rate in biofilm accumulation could be an imbalance in salivary flow due to scarred mucosa as well as saliva properties and composition. Yet, to our knowledge there has been no reports of significant differences in salivary flow, mucosa hydration, pH-value and buffer capacity of EB patients in comparison to a healthy control [33, 34]. Thus, it is not known yet and should be within the scope of future research if these factors influence biofilm accumulation in EB patients.

In the present study, EB patients had an average of 6.9 teeth extracted during therapy in intubation anesthesia, accounting for 32% of their existing teeth, compared to just 1% in the control group. In one case, a total of 23 teeth were extracted from a 17-year-old girl with DEB, illustrating the extent of quality of life impairment at a young age and highlighting the severe impact of EB on dental health [16]. The frequent need for extractions in EB patients is likely due to the advanced stage of dental caries due to late first visits to the dentist, therefore a lack of knowledge in dental prevention. In severe cases, dentists are unable to perform conservative dental treatments effectively on an outpatient basis, as multiple treatment attempts can iatrogenically induce new oral mucosal lesions in these patients. Therefore, if possibilities of preventive and non-invasive measures have been exhausted, fewer treatment appointments and a multidisciplinary approach could be particularly advantageous for EB patients [35]. Furthermore, it is generally not possible to take intraoral or bitewing radiographs of EB patients, especially when microstomia is present. So, the diagnosis must be made using panoramic x-rays, which have a lower sensitivity and specificity in caries detection [36, 37]. The fragility of the oral mucosa and the complications associated with performing dental procedures on EB patients often necessitate extractions as a primary treatment modality. After tooth extraction, the above-mentioned factors also impede the prosthetic restauration of patients with DEB. Mucosa-supported constructions are not an option due to the injury-prone, fragile oral mucosa. Instead, there are isolated case reports of dental implant treatments [38–40]. 

Additional documented symptoms in this study, such as microstomia and ankyloglossia, were prevalent among all listed DEB patients, which is in accordance with previously published literature [19, 41]. These conditions further hamper oral hygiene routines and dental treatments, contributing to the overall poorer oral health observed in the EB group. Disadvantages such as microstomia and ankyloglossia are difficult to prevent. However, guided physiotherapy training, i.e. carefully opening the mouth with the help of assistive devices such as stacked wooden spatulas [42] or acrylic cones [11] while taking care not to hurt the mucosa, would be a further option.

Additionally, all five examined JEB patients showed signs of enamel hypoplasia clinically similar to amelogenesis imperfecta (in x-ray, as well as documentation). A direct association between JEB and amelogenesis imperfecta has as well been described in the literature [7, 43]. One patient out of the JEB group showed severe external crown resorption of the premolars [28].

All in all, this leaves the following assumptions: (i) even though healthy patients also exhibited poor oral hygiene, the extent of fillings and tooth extractions apparently could be prevented by dental supervision and prevention programs, i.e. regular reminding of oral hygiene, low-sugar diet and regular application of fluorides, (ii) the first dental appointment of EB patients is taking place far too late in life, entailing advanced dental destruction; and (iii) it can be hypothesized that earlier and more informative dental care might help prevent or at least postpone severe oral complications in EB patients. Future prospective studies are needed to evaluate the impact of such preventive strategies on oral health outcomes in this population. Above all, it is important to encourage parents of EB patients to visit the dentist before their child’s first tooth erupts and to participate in prevention programs such as regular check-ups and fluoridation [10]. Furthermore, close interdisciplinary cooperation is important to provide patients with all the options for a pain-free and safe treatment [28].

### Limitations and future directions

The retrospective design of the present study and the reliance on existing medical records and panoramic x-rays are limitations that may affect the comprehensiveness of the data. Moreover, since the University Medical Center Freiburg is internationally listed as an EB center, most of the patients were treated only once in intubation anesthesia at the Medical Center, presumably at their worst dental situation, before returning to their home country. Thus, in many international patient cases it is not possible to trace how the further treatment, in particular preventive programs, took place. As the examined cohort originates solely from a specialized EB center, the findings may overestimate the treatment burden typically observed in EB patients managed in community care settings. However, our results are in line with a case-control study including a smaller patient number of 10 EB patients to be under medical supervision at the pediatric dentistry clinic and at the Dermatology Service from the same hospital and 10 controls [44]. In that case control study, EB patients revealed a significantly higher DMFT index of 5 compared to controls with 3 by median and a higher intake of soft food [44]. In contrast, another comparative multicenter study investigating 42 EB and control children each found no difference in terms of enamel defects and caries but gingival inflammation not related to plaque in EB patients, of which 90.2% brushed their teeth at least once a day despite the pain [45]. Additionally, the small sample size in general and the fact, that there were no patients with the Kindler EB type may limit the generalizability of the findings. Furthermore, the medical records contained no information on periodontal characteristics. Future studies should include a larger and more diverse cohort of EB patients to provide a more comprehensive understanding of their oral health challenges. Moreover, the assessment of oral hygiene was based solely on written documentation and therefore solely reflected the judgment of the treating clinicians. Due to the retrospective nature of this study, more detailed information was not available, which may have introduced a certain degree of observer bias here. Nevertheless, to the best of our knowledge the present study is the one with the biggest sample size yet including clinical data from dental treatments of EB patients. Moreover, longitudinal and prospective studies tracking oral health outcomes over time would offer valuable insights into the progression of dental issues in EB patients and the effectiveness of various treatment protocols.

## Conclusion

This study highlights the significant oral health challenges faced by patients with Epidermolysis bullosa and underscores the need for specialized dental care protocols tailored to the unique needs of EB patients. Addressing these challenges through targeted interventions and comprehensive dental care strategies is crucial to improving the oral health and overall quality of life for individuals with EB.

## Data Availability

The datasets used and/or analyzed during this study are available from the corresponding author on reasonable request.

## Abbreviations

EB: Epidermolysis bullosa
EBS: Epidermolysis bullosa simplex
JEB: Epidermolysis bullosa junctionalis
KEB: Kindler epidermolysis bullosa
DEB: Epidermolysis bullosa dystrophica
RDEB: Recessive epidermolysis bullosa dystrophica
DDEB: Dominant epidermolysis bullosa dystrophica
DMFT/dmft: Number of decayed (d; D), missing (m; M) and filled (f; F) teeth
API: Approximal plaque index
SBI: Sulcus bleeding index
